# *Portulaca oleracea* polysaccharide alleviates obesity in mice with long-term high-fat diet by regulating gut microbiota and metabolites

**DOI:** 10.3389/fnut.2026.1759556

**Published:** 2026-02-27

**Authors:** Qiang Fu, Chenglin Zhi, Siyi Cai, Zijian Li, Hui Luo, Hongying Gao, Elvis Agbo, Xiaoliu Huang, Yushan Huang

**Affiliations:** 1 College of Basic Medical Sciences, Jinggangshan University, Ji’an, China; 2 Jiangxi Province Key Laboratory of Organ Development and Epigenetics, Clinical Medical Research Center, Affiliated Hospital of Jinggangshan University, Medical Department of Jinggangshan University, Ji’an, China; 3 Jiangxi Engineering Laboratory of Zebrafish Modeling and Drug Screening for Human Diseases, Key Laboratory of Jiangxi Province for Biological Invasion and Biosecurity, College of Life Sciences, Jinggangshan University, Ji’an, China; 4 Center for Evidence Based Medical and Clinical Research, First Affiliated Hospital of Gannan Medical University, Ganzhou, China

**Keywords:** anti-obesity, gut microbiota, metabolites, mice, *Portulaca oleracea* polysaccharide

## Abstract

**Background:**

Obesity is closely linked to gut microbiota dysbiosis and metabolic disorders. *Portulaca oleracea* polysaccharide (POP) has potential metabolic benefits, but its effects and mechanisms against obesity remain unclear. This study aimed to investigate the ameliorative effects of POP on high-fat diet (HFD)-induced obesity in mice.

**Methods:**

C57BL/6J mice were fed an HFD supplemented with 3.2% POP for 17 weeks. Obesity-related parameters, gut microbiota, and serum metabolomics were analyzed.

**Results:**

POP significantly reduced obesity, improved lipid profiles and glucose homeostasis, increased gut microbiota diversity, and normalized the Firmicutes/Bacteroidetes ratio. It modulated several key gut microbiota genera and altered metabolites including LacCer (d18:1/12:0) and N-(4,7-Dihydroxy-8-Methyl-2-Oxo-2H-Chromen-3-Yl)-2,2-Dimethylchromane-6-Carboxamide (NDC), which strongly correlated with obesity-related indices.

**Conclusion:**

POP may improve HFD-induced obesity by regulating gut microbiota and host metabolism. These results provide a theoretical basis for POP as a potential functional component against obesity.

## Introduction

*Portulaca oleracea*, a plant used both as food and medicine, is known as “global panacea” and “vegetable for long life” (1–3). It is rich in functional compounds such as polysaccharides, flavonoids, terpenoids, and organic acids. POP, the main active components, exhibit effects such as hypoglycemia and lipid regulation (4). In recent years, animal studies and clinical trials have demonstrated the potential anti-obesity effects of *P. oleracea*. Adding 10% *P. oleracea* powder to a HFD in mice inhibited weight gain, reduced body fat and blood glucose levels by upregulating the expression of peroxisome proliferator-activated receptor (PPAR)-α, glucose transporter (GLUT) 4, and PPAR-*γ* proteins (5). A double-blind, randomized controlled clinical trial in Iran showed that a mixed supplement containing *P. oleracea*, *Plantago psyllium*, and peanut oil effectively reduced body weight and appetite in overweight and obese individuals (6). *P. oleracea* significantly lowers fasting blood glucose and lipid levels in patients with diabetes or hyperlipidemia (7). Given the complex compounds in *P. oleracea*, it is necessary to study the anti-obesity properties of its main active component, polysaccharides. However, current understanding of the anti-obesity effects and their underlying mechanisms of polysaccharides, the major active component of *P. oleracea*, remains limited, which has hindered the full exploitation of the plant’s resources.

Obesity has become a global public health issue, resulting from long-term imbalance between energy intake and expenditure, leading to excessive accumulation of adipose tissue (8, 9). This condition is closely linked to chronic diseases such as insulin resistance, non-alcoholic fatty liver disease (NAFLD), and metabolic syndrome, reducing quality of life (10, 11). Increasing studies show that obesity is significantly influenced by gut microbiota (12, 13). Studies showing that germ-free mice transplanted with feces from HFD-fed mice developed obesity-related characteristics confirm that intestinal dysbiosis is a key factor in HFD-induced obesity (14, 15). Notably, as an important dietary fiber, plant polysaccharides can exert their health-promoting effects by selectively modulating the gut microbiota (16). For instance, *Phyllanthus emblica* L. polysaccharides can alleviate obesity symptoms by regulating lipid metabolism and the gut microbiota in HFD-fed mice (17). This inspires us to investigate whether gut microbiota and metabolism are associated with the anti-obesity effects of POP. Therefore, the main objective of this study is to confirm the interaction between POP and gut microbiota, as well as its mechanism of action on HFD-induced obesity.

## Materials and methods

### Materials

*Portulaca oleracea* polysaccharide was prepared by Lanzhou Wotelaisi Biotechnology Co., Ltd. using a method modified from Ning et al. (18) with further optimization (e.g., a solid-liquid ratio of 1:7 and extraction at 90–95 °C for 2 h). Animal feeds MD12032 (45% kcal fat), MD12031 (10% kcal fat), and MD12032 containing 3.2% (wt/wt) POP were prepared by Jiangsu Meidison Biomedical Co., Ltd., based on our previous research (4). The dietary components are listed in Supplementary Table S1.

### Animals and diet

Thirty 4/5 week-old male C57BL/6J mice were purchased from Hunan Silaike Jingda Experimental Animal Co., Ltd. Mice were housed in the Experimental Animal Center of Jinggangshan University (SYXK<Jiangxi>2023-0009) under specific pathogen-free (SPF) conditions at 23 ± 1 °C, 50–60% relative humidity, with a 12/12 light/dark cycle, and free access to food and water. After one week of adaptive feeding, all animals were randomly divided into three groups (*n* = 10 each): normal diet group (Con) fed MD12031, HFD group fed MD12032, and POP group fed MD12032 containing 3.2% POP, for 17 weeks. Body weight and food intake were recorded weekly. At the end of the experiment, mice were anesthetized with isoflurane by inhalation using a small animal ventilator (oxygen flow rate: 0.5–0.7 L/min; isoflurane concentration: 1.0–1.5%), followed by blood collection via orbital enucleation. Liver, perirenal fat, epididymal fat, inguinal fat, and brown fat tissue were dissected, weighed, and stored at −80 °C. All experimental protocols were approved by the Ethics Committee of Jinggangshan University Experimental Animal Center [(2023) Ethical Approval No. (159)]. All procedures involving animals were performed in accordance with the NIH Guide for the Care and Use of Laboratory Animals (8th edition), and the Guidelines for the Care and Use of Laboratory Animals issued by the Ministry of Science and Technology of China.

### Oral glucose tolerance test (OGTT)

At week 16, mice were fasted for 12 h and then orally administered glucose at a dose of 2 g/kg body weight. Glucose concentrations in tail blood were measured at 0, 15, 30, 60, 90, and 120 min using a handheld blood glucose meter (Yuyue 580, Yuyue Medical Equipment Co., Ltd., China).

### Serum biochemical analysis

Blood samples were collected by orbital exsanguination, left at room temperature for 4 h, and then centrifuged to obtain serum. Levels of TC, TG, high-density lipoprotein cholesterol (HDL-C), and LDL-C in serum were measured using an automatic biochemical analyzer (Dimension RxL Max, Siemens, United States) according to the manufacturer’s instructions.

### Histological analysis

Liver, inguinal white adipose tissue (iWAT), and brown adipose tissue fixed in 4% paraformaldehyde were dehydrated in gradient ethanol, embedded in paraffin, sectioned, and stained with hematoxylin and eosin (H&E) (19). Fresh liver tissue was frozen-sectioned and stained with Oil Red O according to the kit instructions (Beyotime C0158S#, China). Images were acquired using an Olympus BX53 microscope (Olympus, Tokyo, Japan).

### Gut microbiota analysis

Fecal samples collected from the colon were sent to OE Biotech Co., Ltd. (Shanghai, China) for 16S rRNA gene sequencing. Briefly, total genomic DNA was extracted using the MagPure Soil DNA LQ Kit (Magan) according to the manufacturer’s instructions. The V3-V4 hypervariable regions (343F 5′-TACGGRAGGCAGCAG-3′ and 798R 5′-AGGGTATCTAATCCT-3′) of the prokaryotic 16S rRNA were amplified for bacterial diversity analysis. Raw data were processed by cutting off primer sequences using Cutadapt software. Qualified paired-end raw data were subjected to quality filtering, denoising, splicing, and chimera removal using the DADA2 algorithm with default parameters in QIIME 2 (V2020.11) to obtain final valid data for bioinformatics analysis.

### Untargeted metabolomics in fecal samples

Thirty milligrams of fecal samples were weighed out and mixed with 400 μL of methanol-water (v:v = 4:1, containing L-2-chlorophenylalanine at 4 μg/mL). The mixture was precooled at −40 °C for 2 min, ground for 2 min (60 Hz), subjected to ultrasonic extraction in an ice-water bath for 10 min, and then incubated at −40 °C for 30 min, followed by centrifugation at 12,000 rpm at 4 °C for 10 min. Three hundred microliters of the supernatant was collected and dried under nitrogen. The residue was reconstituted with 300 μL of methanol-water (v:v = 1 : 4), vortexed for 30 s, subjected to ultrasonic treatment in an ice-water bath for 3 min, and incubated at −40 °C for 2 h. The reconstituted extract was centrifuged at 12,000 rpm at 4 °C for 10 min. One hundred and fifty microliters of the supernatant was filtered through a 0.22 μm organic phase syringe filter and stored at −80 °C until LC-MS/MS analysis. Quality control (QC) samples were prepared by mixing equal volumes of extracts from all fecal samples. The insertion frequency of QC samples was set as follows: once before sample detection, once every 7 samples during the detection process, and twice consecutively after the completion of all sample detection.

The UPLC-MS/MS analysis was performed using an ACQUITY UPLC I-Class plus (Waters Corporation, Milford, United States)/Thermo QE HF (Thermo Fisher Scientific, Waltham, MA, United States) system with an ACQUITY UPLC HSS T3 (100 mm × 2.1 mm, 1.8 μm) column. It was equipped with a heated electrospray ionization (ESI) source (Thermo Fisher Scientific, Waltham, MA, United States), which was used for analyzing metabolic profiles in both positive and negative ESI modes. The gradient elution system consisted of (A) water (containing 0.1% formic acid, v/v) and (B) acetonitrile (containing 0.1% formic acid, v/v), with the following gradient program: 5% B at 0.01 min; 5% B at 2 min; 30% B at 4 min; 50% B at 8 min; 80% B at 10 min; 100% B at 14 min; 100% B at 15 min; 5% B at 15.1 min; and 5% B at 16 min. The flow rate was 0.35 mL/min, the column temperature was maintained at 45 °C, and the injection volume was 2 μL. The mass range was set from 70 *m*/*z* to 1,050 *m*/*z*, with a resolution of 70,000 for full-scan MS (MS^1^) and 17,500 for MS/MS (MS^2^) scans. Collision energies of 10, 20, and 40 eV were applied for MS/MS fragmentation. The operating parameters of the mass spectrometer were as follows: spray voltage: 3800 V (+) and 3,000 V (−); sheath gas flow rate: 35 Arb; auxiliary gas flow rate: 8 Arb; capillary temperature: 320 °C; auxiliary gas heater temperature: 350 °C; and S-lens RF level: 50 (20). Information of internal standards, solvents, and reagents are listed in Supplementary Table S2.

Raw data were processed for baseline filtering, peak identification, integration, retention time correction, peak alignment, and normalization using Progenesis QI V2.3 (Nonlinear Dynamics, Newcastle, United Kingdom), with key parameters set as follows: precursor ion tolerance of 5 ppm, product ion tolerance of 10 ppm, and product ion threshold of 5%. Compound identification was based on precise mass-to-charge ratio (*m*/*z*), secondary fragments, and isotopic distribution using The Human Metabolome Database (HMDB), Lipidmaps (V2.3), Metlin, and self-built databases (LuMet-Animal3.0). Preprocessed data were analyzed on the Oebiotech Cloud platform^1^. The matrix was imported into R for principal component analysis (PCA) to observe the overall distribution of samples and the stability of the analysis process. Orthogonal partial least squares-discriminant analysis (OPLS-DA) was used to identify differential metabolites between groups. Metabolites with *p* < 0.05 and VIP > 1 were selected as differential metabolites. Differential metabolites were further subjected to Kyoto Encyclopedia of Genes and Genomes (KEGG) pathway^2^ enrichment analysis.

### Statistical analysis

Data are presented as means ± SEM and analyzed using GraphPad Prism 8.1.0 (GraphPad Software, United States). One-way analysis of variance (ANOVA) was used to determine significant differences. A *p*-value <0.05 or <0.01 was considered statistically significant between groups. Correlation analysis of obesity-related parameters with intestinal flora and metabolites was performed using Spearman correlation analysis and Euclidean distances-based redundancy analysis (RDA).

## Results

### Long-term POP supplementation alleviated body weight gain in HFD-fed mice

**Figure 1:**
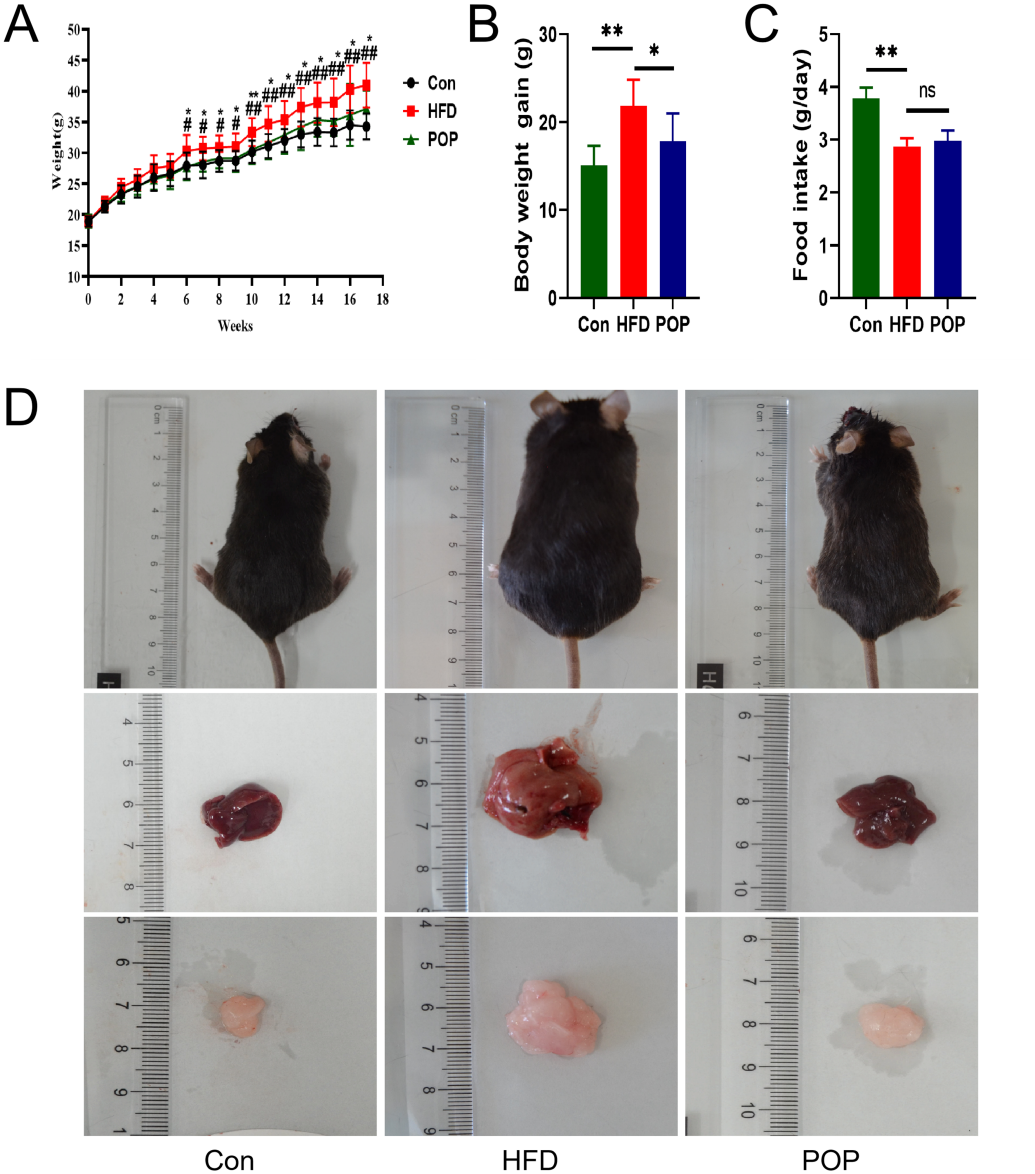
Long-term supplementation with POP affects body weight gain in HFD-fed mice. **(A)** Body weight changes over 17 weeks. ^#^*p* < 0.05, ^##^*p* < 0.01 vs. con group; *p < 0.05, **p < 0.01 vs. HFD group. **(B)** Body weight gain. **(C)** Food intake. **(D)** Representative photographs of mouse body shape (upper), liver (middle), and iWAT (lower) after POP supplementation. One-way ANOVA was used to determine significant differences. Data are presented as mean ± SD (*n* = 10 mice per group), *p < 0.05, **p < 0.01.

To evaluate the anti-obesity effect of POP, C57BL/6J mice were fed a normal diet (Con), HFD, or HFD with POP (POP) for 17 weeks. Notably, body weight in the HFD group was significantly higher than that in the Con group after 6 weeks of HFD feeding and remained higher until the end of the experiment (Figure 1A). However, POP supplementation significantly reduced body weight gain in HFD-fed mice (Figure 1A). Additionally, POP supplementation decreased total body weight gain induced by HFD (Figure 1B), independent of food intake (Figure 1C). These results indicate that the effect of POP on body weight was not related to changes in food consumption. Representative images of the mice’s overall appearance, liver, and iWAT are shown in Figure 1D, demonstrating significant improvement in liver steatosis and fat accumulation.

### Long-term POP supplementation prevented histopathological changes in adipose tissue of HFD-fed mice

**Figure 2:**
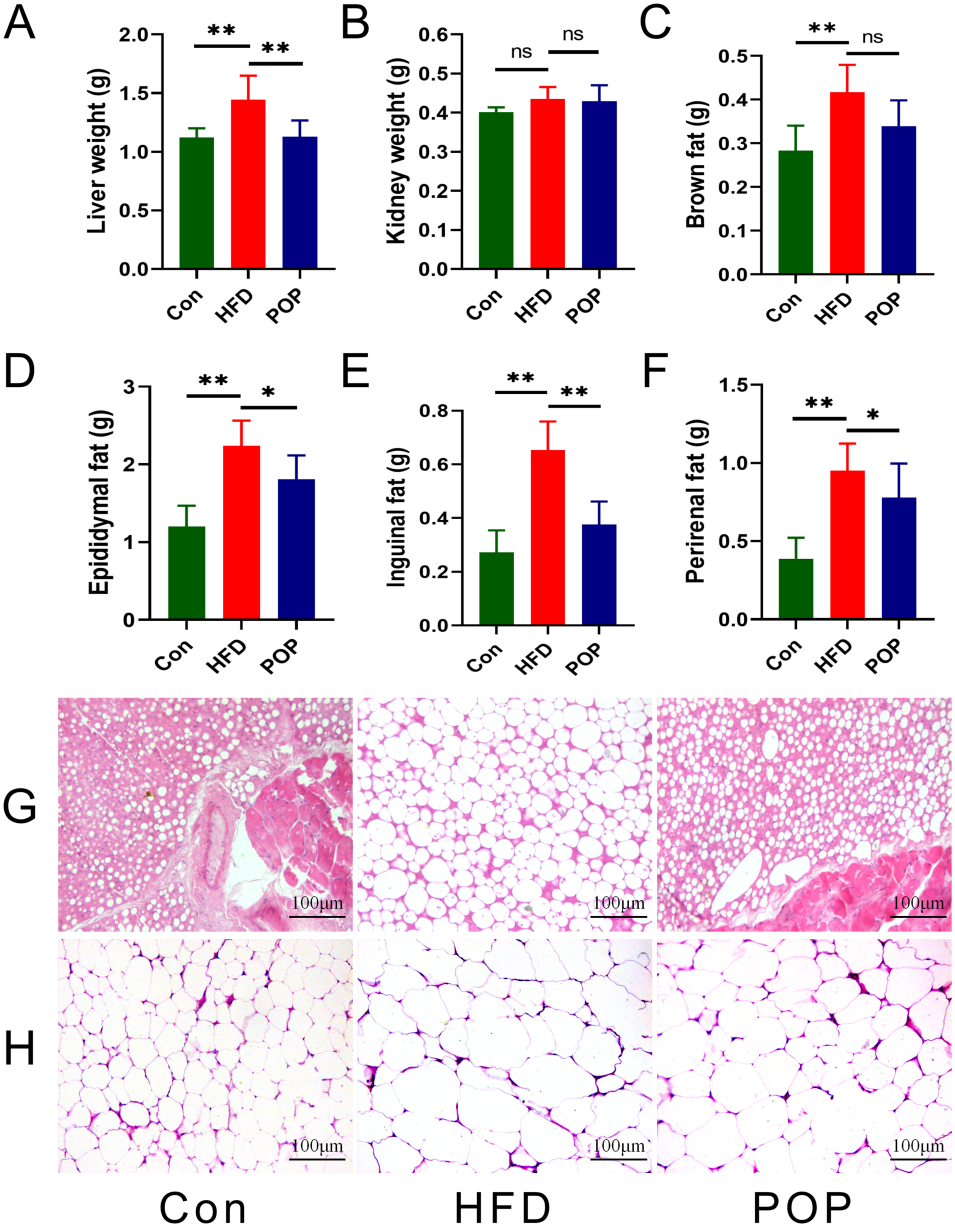
Long-term POP supplementation alleviates white adipose tissue hypertrophy in HFD-fed mice. **(A)** Liver weight, **(B)** kidney weight, **(C)** brown fat weight, **(D)** epididymal fat (eWAT) weight, **(E)** inguinal fat (iWAT) weight, **(F)** perirenal fat (pWAT) weight. Representative H&E staining of **(G)** brown adipose tissue and **(H)** inguinal fat tissue. One-way ANOVA was used to determine significant differences. Data are presented as mean ± SD (*n* = 10 mice per group), *p < 0.05, **p < 0.01 vs. HFD group.

Previous studies have shown that long-term HFD feeding may cause histopathological changes in adipose tissue (15). In this study, we found hypertrophy in liver and adipose tissues in HFD-fed mice. However, POP supplementation significantly reduced the weights of liver, white adipose tissue (epididymal fat, eWAT; inguinal fat, iWAT; and perirenal fat, pWAT) in HFD-fed mice. Meanwhile, H&E staining analysis showed that brown adipocytes in HFD mice supplemented with POP were smaller, with reduced lipid droplet size (Figure 2G). Additionally, POP supplementation significantly reduced the cell size of iWAT in HFD-fed mice (Figure 2H).

### Long-term POP supplementation prevented metabolic abnormalities and liver histopathological changes in HFD-fed mice

**Figure 3:**
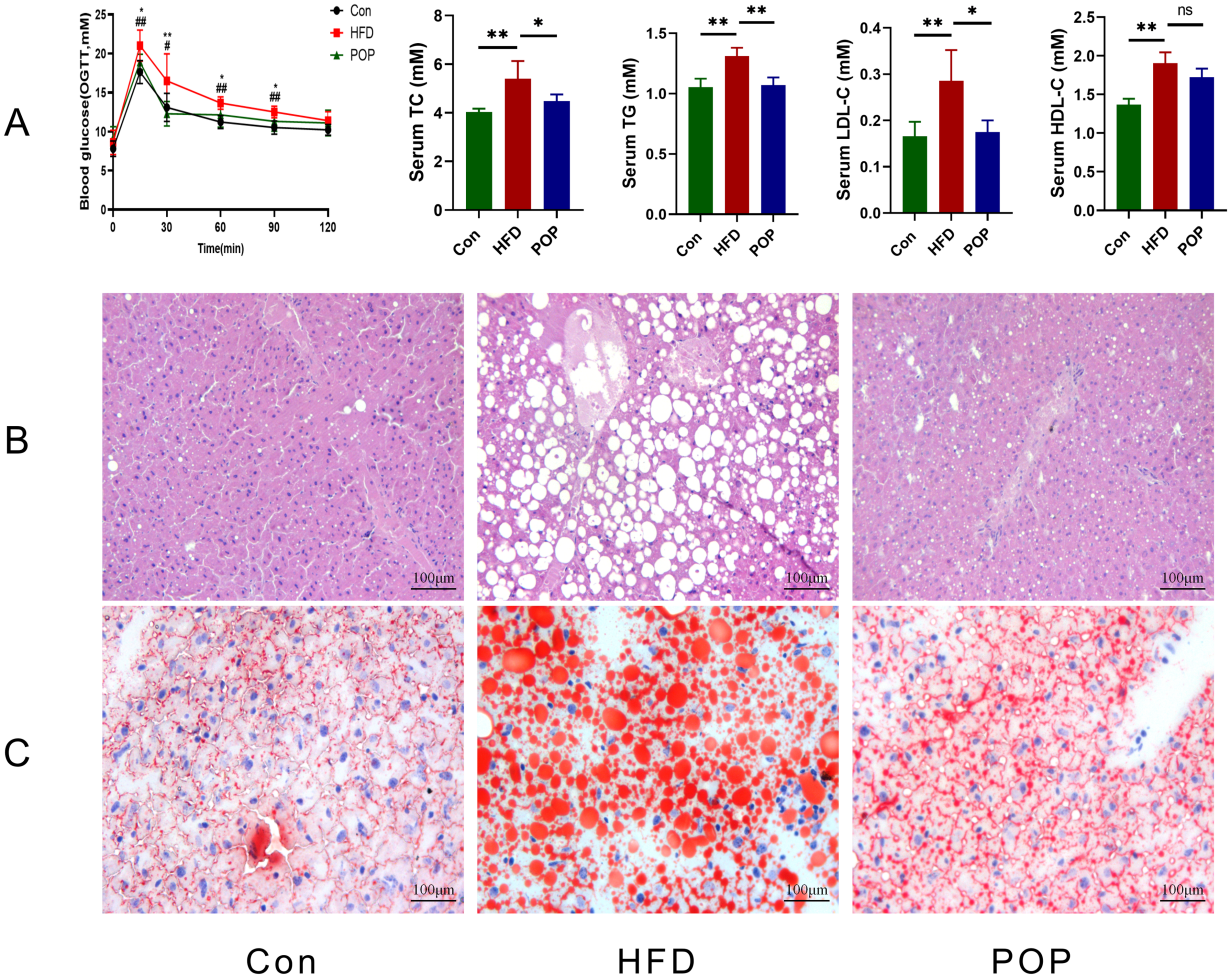
Long-term POP supplementation improves lipid and glucose homeostasis in HFD-fed mice. **(A)** OGTT and serum concentrations of LDL-C, TG, HDL-C, and TC. **(B)** H&E-stained and **(C)** oil red O-stained liver tissues. One-way ANOVA was used to determine significant differences. Data are presented as mean ± SD (*n* = 10 mice per group), *p < 0.05, **p < 0.01 vs. HFD group.

Abnormalities in glucose and lipid homeostasis are common metabolic disorders in obese patients (10). We further analyzed oral glucose tolerance (OGTT) and serum lipid levels. Results showed that the blood glucose response in the HFD group exceeded that in the Con group throughout the OGTT, while POP treatment improved glucose tolerance in OGTT. Serum biochemical indices including TG, TC, LDL-C, and HDL-C were measured. As shown in Figure 3A, HFD significantly increased TG, TC, LDL-C, and HDL-C levels. POP intervention reversed this trend, indicating that POP can improve HFD-induced changes in lipid levels. Additionally, histological evaluation of liver tissue showed hepatocyte disorder and numerous lipid droplet vacuoles in HFD-fed mice, which were reversed by POP intake (Figures 3B,C).

### POP regulated gut microbiota composition in HFD-fed mice

**Figure 4:**
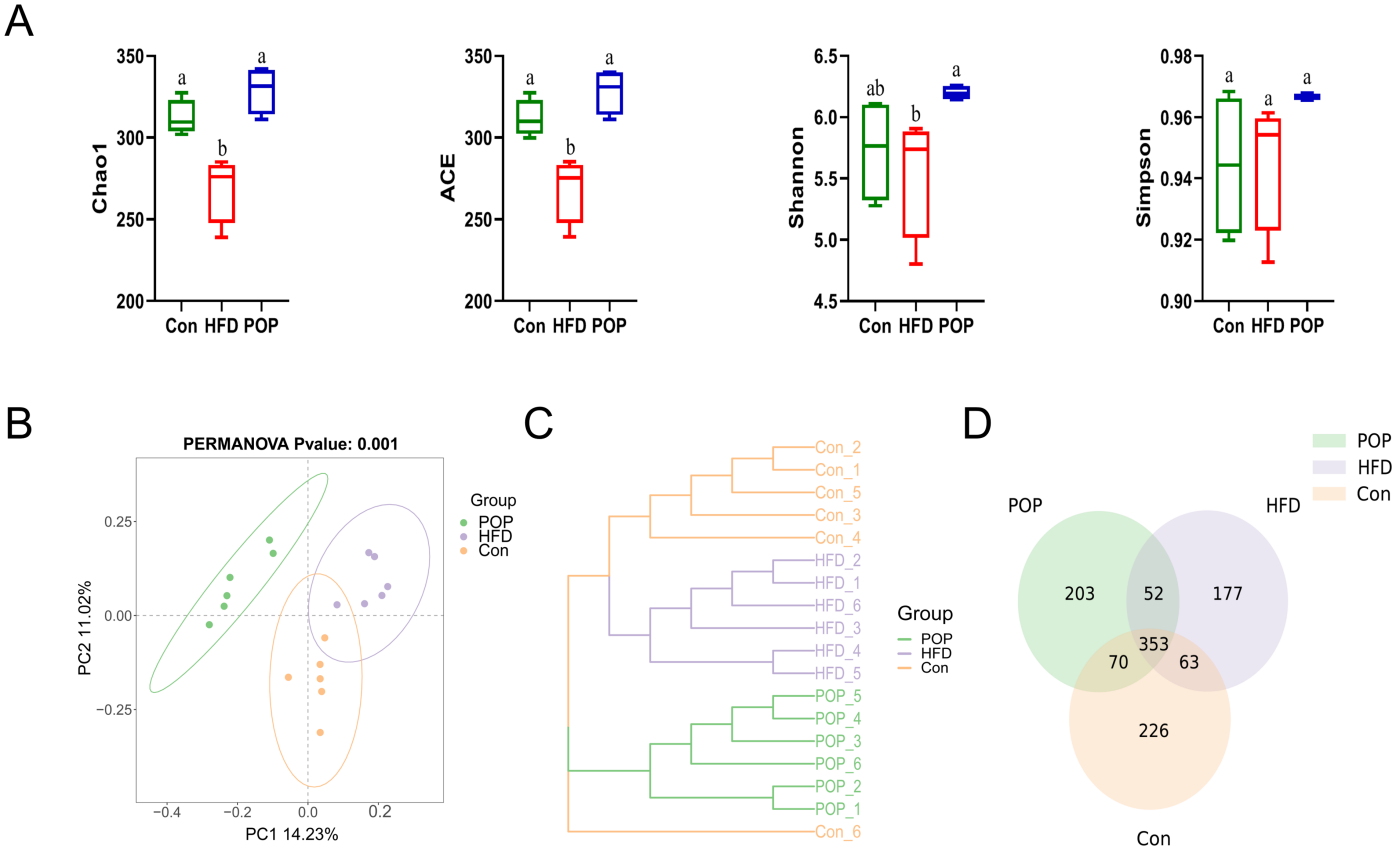
Effects of long-term POP supplementation on gut microbiota diversity in HFD-fed mice. **(A)** Chao1 Index, ACE Index, Shannon Index, and Simpson Index. **(B)** PCoA of gut microbiota. **(C)** UPGMA cluster results of unweighted UniFrac distances. **(D)** Venn diagrams at the OTU level. One-way ANOVA was used to determine significant differences. Data are presented as mean ± SD (*n* = 10 mice per group); different lowercase letters on bar charts indicate statistical differences between groups.

Considering that HFD feeding leads to intestinal dysbiosis, we detected gut microbiota structure by 16S rRNA analysis. Figure 4A shows that the *α*-diversity indices Chao1 and ACE were significantly lower in the HFD group compared to the Con and POP groups, while the Shannon index in the POP group was significantly higher than in the HFD group. Principal coordinate analysis (PCoA) showed significant differences in gut microbiota composition among different diet groups (Figure 4B), indicating important changes in gut microbial profiles after POP supplementation. Unweighted pair group method with arithmetic mean (UPGMA) analysis based on the binary Jaccard algorithm also confirmed these differences between groups (Figure 4C). The number of unique and shared bacteria at the OTU level is listed in Figure 4D. The Con group had 226 unique bacteria, while the HFD and POP groups had 177 and 203, respectively, indicating that HFD and POP altered gut microbiota.

**Figure 5:**
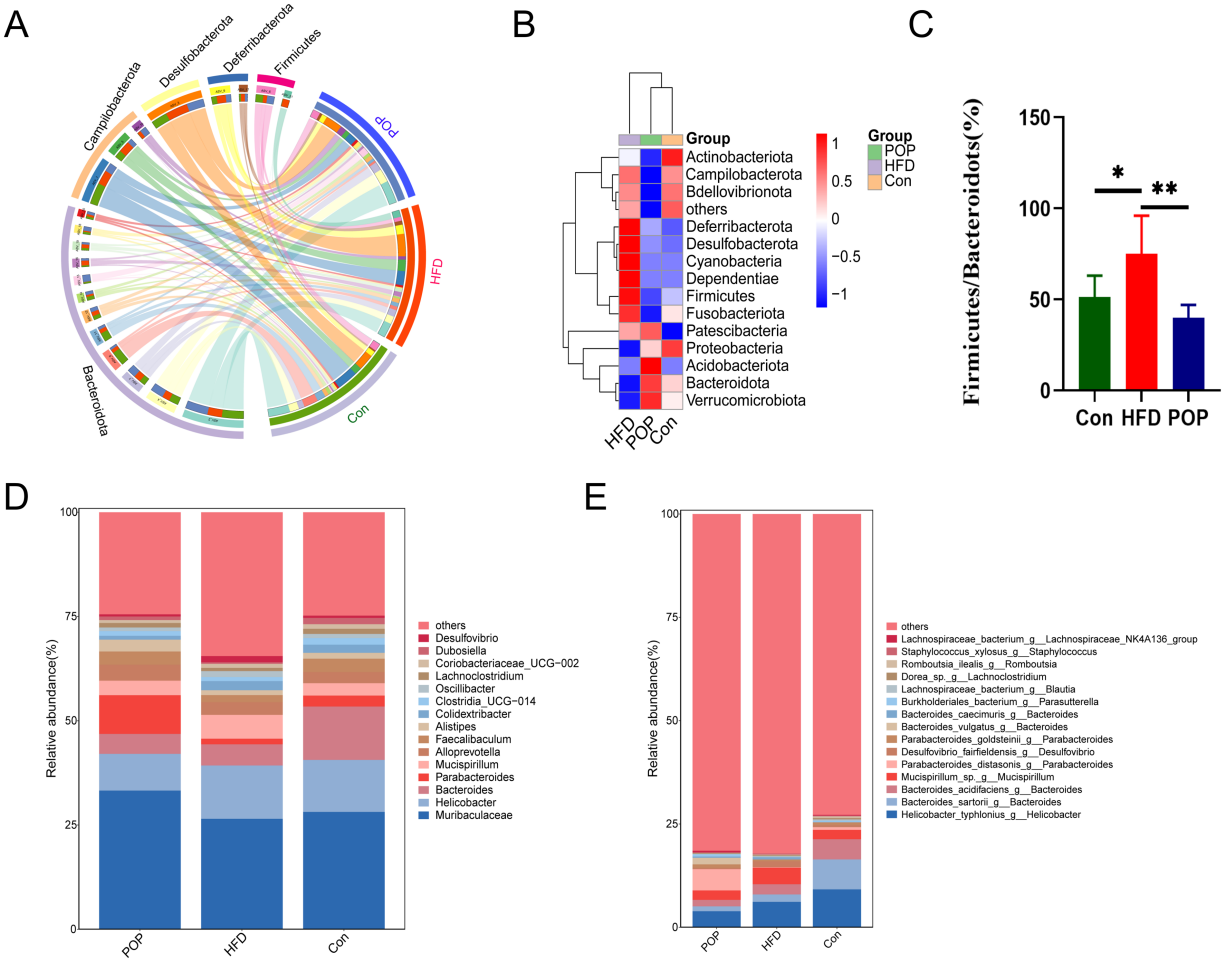
Long-term POP supplementation reshapes gut microbiota in HFD-fed mice. **(A)** Circo’s graphs showing the distribution proportion of dominant gut microbiota in each group at the phylum level. **(B)** Heatmap of dominant phyla composition. **(C)** The *F*/*B* ratio. **(D)** Changes in gut microbiota at the genus level. **(E)** Changes in gut microbiota at the species level. One-way ANOVA was used to determine significant differences. Data are presented as mean ± SD (*n* = 10 mice per group), *p < 0.05, **p < 0.01 vs. HFD group.

Next, we further studied the composition and changes of gut microbiota at the phylum and genus levels. Results showed five dominant bacterial phyla at the phylum level (Figures 5A,B). HFD increased the relative proportions of Firmicutes, Desulfobacterota, Deferribacterota, and Campilobacterota, while decreasing the relative proportion of Bacteroidota. Conversely, these changes were reversed by POP intervention. Additionally, HFD led to an increase in the *F*/*B* ratio (Figure 5C), a common gut microbiota feature in insulin-resistant and obese individuals. Interestingly, long-term POP supplementation reduced the *F*/*B* ratio in HFD-fed mice to a level similar to the Con group.

At the genus level, community bar charts showed the top 15 gut microbiota genera in each group, with *Muribaculaceae*, *Helicobacter*, *Bacteroides*, *Parabacteroides*, and *Mucispirillum* being the most dominant (Figure 5D). In particular, compared to the Con group, HFD significantly increased the proportions of *Helicobacter* and *Mucispirillum*, while significantly decreasing the abundances of *Muribaculaceae* and *Parabacteroides*. Notably, POP supplementation enriched the abundances of *Muribaculaceae* and *Parabacteroides*, while reducing those of *Helicobacter* and *Mucispirillum*. Figure 5E shows the 15 bacteria with the highest relative abundances at the species level. Compared to the HFD group, POP significantly reduced the abundances of *Helicobacter typhlonius*, *Bacteroides sartorii*, *Bacteroides acidifaciens*, and *Mucispirillum* sp., while increasing the abundance of *Parabacteroides distasonis*. Collectively, these findings provide complementary evidence for the effects of POP on the community and composition of gut microbiota in HFD-induced obese mice.

### POP restored key bacterial genera in gut microbiota

**Figure 6:**
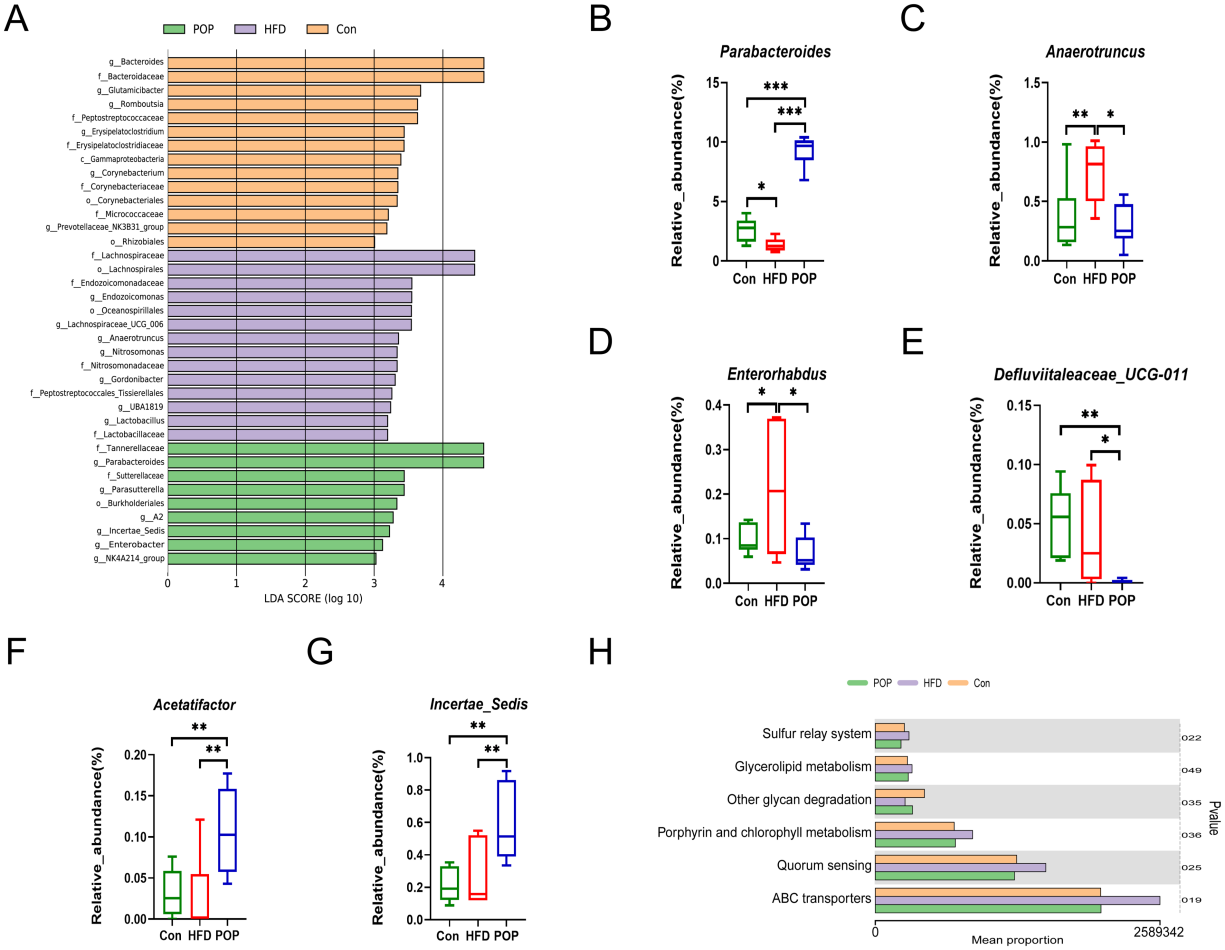
POP regulates key bacterial genera in gut microbiota. **(A)** Barplot of LEfSe analysis (LEfSe, LDA score >3). **(B–G)** Relative abundances of *Parabacteroides* **(B)**, *Anaerotruncus* **(C)**, *Enterorhabdus* **(D)**, *Defluviitaleaceas_UCG-011* **(E)**, *Acetatifactor* **(F)**, and *Incertae_Sedis* **(G)** at the genus level. **(H)** KEGG pathway analysis. One-way ANOVA was used to determine significant differences. *p < 0.05, **p < 0.01, ***p < 0.001 compared between two groups (*n* = 6 samples per group).

Linear Discriminant Analysis Effect Size (LEfSe) is a method for identifying high-dimensional biomarkers and revealing genomic features. We used Linear Discriminant Analysis (LDA) to estimate the impact of characteristic species. The histogram of LDA values (Figure 6A) shows species with LDA scores >3, which were statistically different between groups. Biomarkers in the Con group mainly included *Gammaproteobacteria*, *Corynebacteriales*, *Rhizobiales*, *Bacteroidaceae*, *Peptostreptococcaceae*, *Erysipelatoclostridiaceae*, and *Micrococaceae* and their subclasses. The HFD group was dominated by *Lachnospirales* and *Oceanospirillales*. The POP group mainly included *Burkholderiales*, *Tannerellaceae*, and *Sutterellaceae*. Representative specific bacteria in all groups are further shown in Figures 6B–G. The relative abundances of *Anaerotruncus* and *Enterorhabdus* in the HFD group were significantly higher than those in the Con group. However, POP intervention significantly reduced their abundances (Figures 6C,D). Meanwhile, compared to the Con and HFD groups, the relative abundances of *Parabacteroides*, *Acetatifactor*, and *Incertae_Sedis* in the POP group were significantly increased (Figures 6B,F,G), while *Defluviitaleaceas_UCG-011* was significantly decreased (Figure 6E). Additionally, gut microbiota in the HFD group showed significantly more genes related to sulfur relay system, glycerolipid metabolism, porphyrin and chlorophyll metabolism, quorum sensing (QS), and ABC transporters, but fewer genes related to other glycan degradation (Figure 6H).

### Effects of POP on metabolites

**Figure 7:**
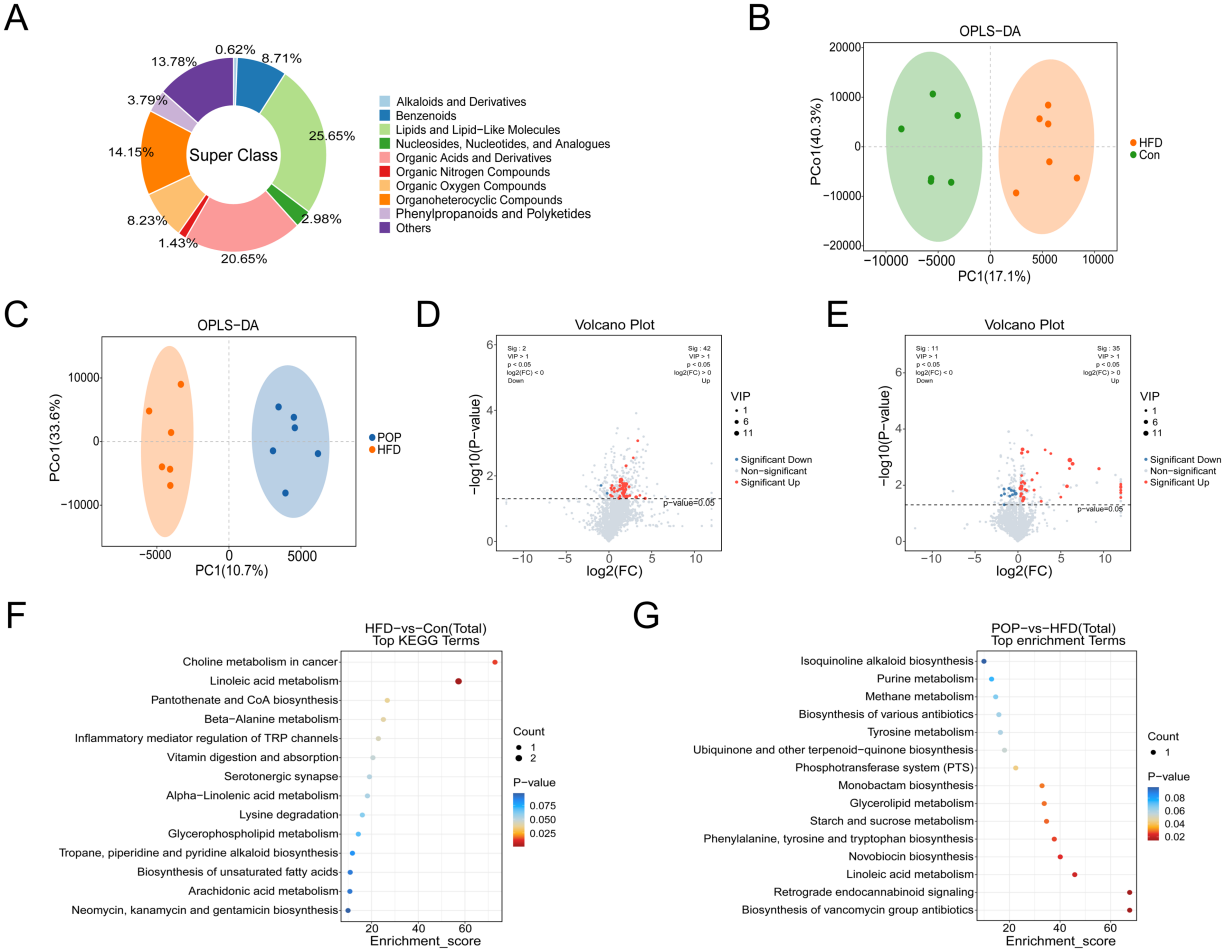
Long-term POP supplementation alters metabolite profiles in HFD-fed mice. **(A)** Classification of identified metabolites. **(B)** OPLS-DA score plots of fecal metabolites in HFD and Con groups. **(C)** OPLS-DA score plots of fecal metabolites in POP and HFD groups. **(D)** Volcano plots of differential metabolites between HFD and Con groups. **(E)** Volcano plots of differential metabolites between POP and HFD groups (*n* = 6 samples per group). Red and blue dots represent significantly upregulated and downregulated metabolites, respectively. The size of the dots denotes the weighted contribution value to the differences. **(F,G)** Pathway analysis of differential metabolites. One-way ANOVA was used to determine significant differences (*n* = 6 samples per group).

To reveal metabolic changes induced by POP, we analyzed the metabolic profiles of colonic contents from mice on normal diet, HFD, and HFD with POP using positive/negative ion LC-MS/MS mode. Metabolite annotation showed that all metabolites mainly belonged to nine categories, including Lipids and Lipid-like Molecules, Organic Acids and Derivatives, Organoheterocyclic Compounds, Benzenoids, etc. (Figure 7A). Partial least squares discriminant analysis (PLS-DA) models were used to detect the relationship between metabolite levels and sample types. Comparisons between groups showed that different dietary patterns and POP intervention led to metabolic differences, with mice in each group clustering separately (Figures 7B,C). Differential analysis identified 44 differential metabolites between the HFD and Con groups, including 42 upregulated and 2 downregulated metabolites (Figure 7D and Supplementary Table S3), and 46 differential metabolites between the POP and HFD groups, including 35 upregulated and 11 downregulated metabolites (Figure 7E and Supplementary Table S4). To reveal the potential functions of differential metabolites, KEGG analysis was performed. The main metabolic pathways between the HFD and Con groups included linoleic acid metabolism, glycerophospholipid metabolism, biosynthesis of unsaturated fatty acids, alpha-linolenic acid metabolism, and arachidonic acid metabolism (Figure 7F and Supplementary Table S5). The main metabolic pathways between the POP and HFD groups included glycerolipid metabolism and linoleic acid metabolism (Figure 7G and Supplementary Table S6).

**Figure 8:**
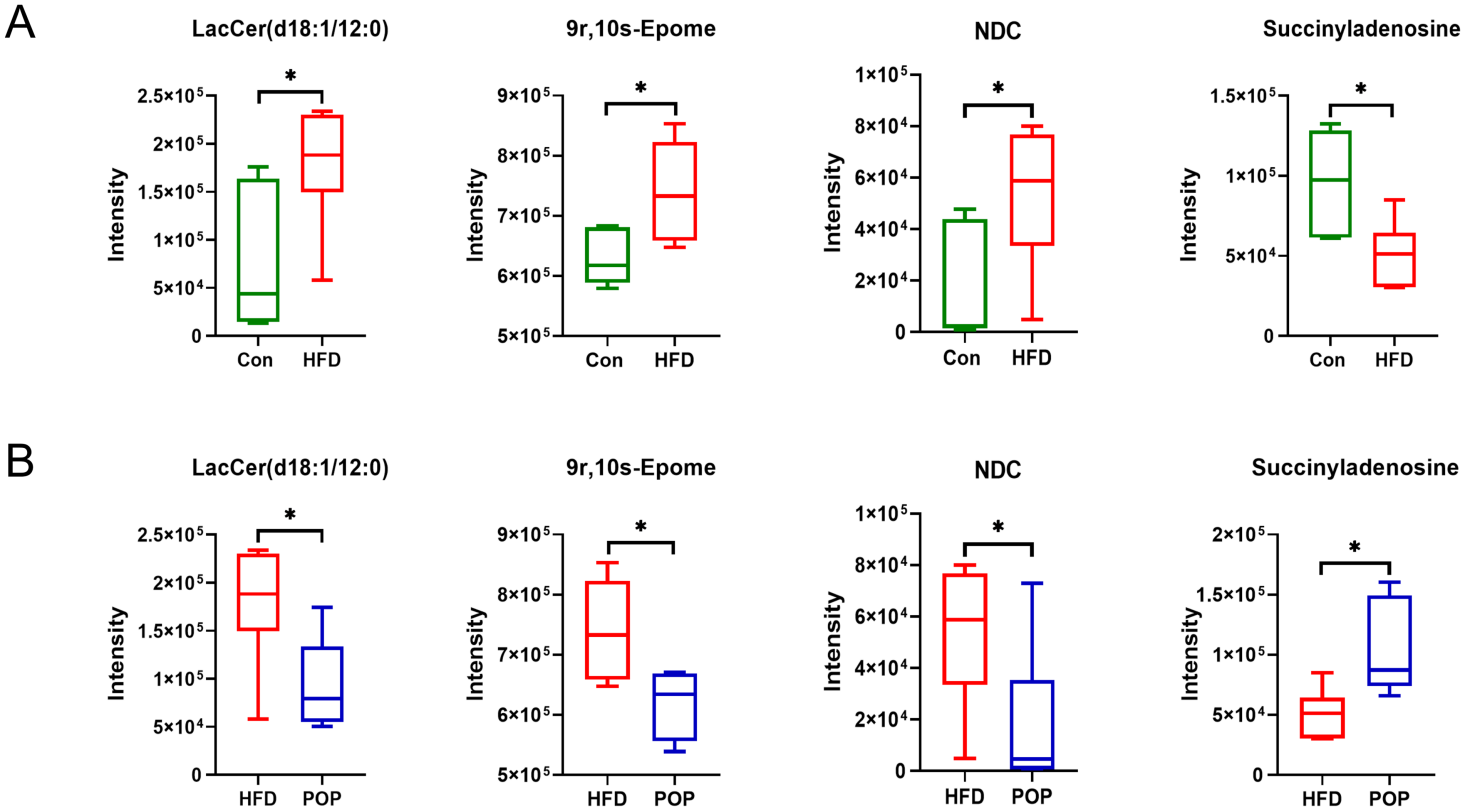
POP regulates key metabolites. **(A)** Key differential metabolites between Con and HFD groups. **(B)** Key differential metabolites between POP and HFD groups. NDC: N-(4,7-Dihydroxy-8-Methyl-2-Oxo-2H-Chromen-3-yl)-2,2-Dimethylchromane-6-Carboxamide. One-way ANOVA was used to determine significant differences. *p < 0.05 compared between two groups (*n* = 6 samples per group).

Moreover, the common differential metabolites among the Con, HFD, and POP groups are presented in Figure 8. Compared to the Con group, 9r,10s-Epome, LacCer (d18:1/12:0), and NDC were significantly increased in the HFD group, while succinyladenosine was significantly decreased (Figure 8A). Long-term POP supplementation reversed these metabolic changes compared to the HFD group (Figure 8B).

### Correlation analysis between gut microbiota, differential metabolites, and obesity-related parameters

**Figure 9:**
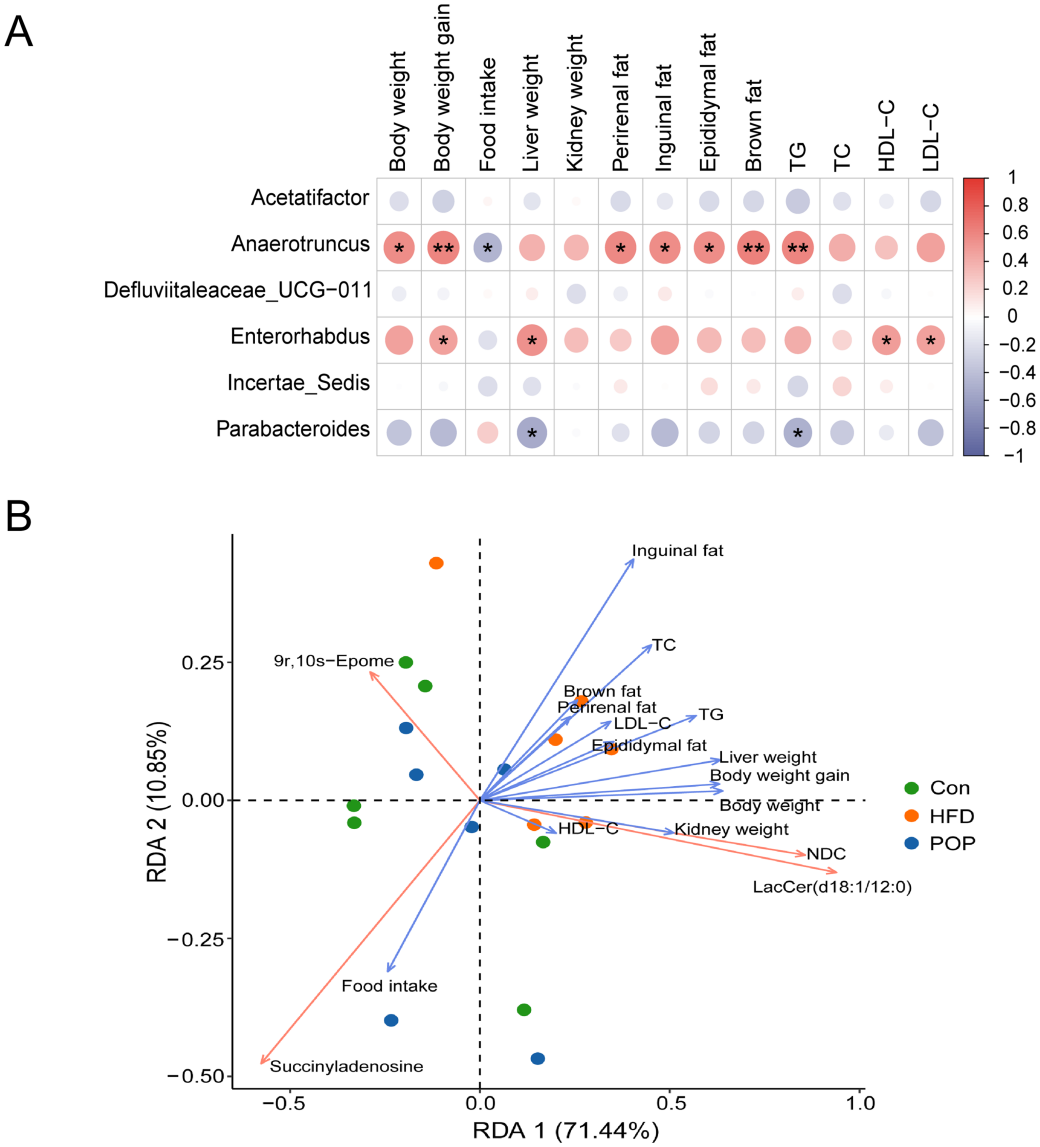
Correlation analysis between obesity-related parameters, gut microbiota, and metabolites. **(A)** Spearman’s correlation heatmap analysis of obesity-associated parameters with six important differential bacterial genera. Red indicates positive correlation, blue indicates negative correlation. The size of the circle represents the significance level. One-way ANOVA was used to determine significant differences. *p < 0.05, **p < 0.01. **(B)** Euclidean distance-based RDA between obesity-related parameters, gut microbiota, and metabolites. Blue arrows represent quantitative obesity-related parameters, and orange arrows represent quantitative metabolites. The length of the arrow indicates the degree of influence of the quantitative index on the microbial community. An acute angle between quantitative indices indicates a positive correlation, while an obtuse angle indicates a negative correlation.

Spearman’s correlation analysis and Euclidean distances-based RDA were used to further explore the interaction between gut microbiota and obesity-related biochemical parameters. As shown in Figure 9A, *Anaerotruncus* was positively correlated with body weight, body weight gain, perirenal fat, inguinal fat, epididymal fat, brown fat, and TG, and negatively correlated with food intake. *Enterorhabdus* was positively correlated with body weight gain, liver weight, HDL-C, and LDL-C. Additionally, the *Parabacteroides* genus was negatively correlated with liver weight and TG.

The RDA results (Figure 9B) showed that succinyladenosine was negatively correlated with body weight, body weight gain, perirenal fat, inguinal fat, epididymal fat, brown fat, TG, TC, LDL-C, liver weight, and kidney weight, and positively correlated with food intake. In contrast, LacCer (d18:1/12:0) and NDC were positively correlated with body weight, body weight gain, perirenal fat, inguinal fat, epididymal fat, brown fat, TG, TC, LDL-C, HDL-C, liver weight, and kidney weight. Meanwhile, 9r,10s-Epome was positively correlated with inguinal fat.

## Discussion

The mechanism of POP’s anti-obesity effect remains poorly understood. Our study provides evidence that long-term POP intake effectively improves HFD-induced obesity and related metabolic disorders. Our data show that POP reduces body weight, liver lipid accumulation, and adipocyte hypertrophy (Figures 1, 2). Adipose tissue and the liver are major endocrine organs involved in obesity development (8). Energy imbalance in obesity leads to adipocyte hypertrophy and lipid deposition in the liver (21, 22). Additionally, abnormal blood glucose and lipid levels are closely associated with obesity syndromes (10). In our previous studies and those of others, POP improved lipid profiles in aging rats and glucose tolerance in diabetic rats (23, 24), consistent with the present findings that POP reduces lipid levels, regulates glucose homeostasis, and inhibits lipid accumulation in the liver of HFD-fed mice.

An increasing consensus highlights gut microbiota as a key factor influencing obesity and related diseases, with its composition and metabolites changing during obesity progression (25, 26). Our results show that POP increases gut microbiota diversity, alters microbial structure, and promotes the growth of beneficial bacteria. Studies have shown that obese individuals exhibit a higher *F*/*B* ratio at the phylum level (12). In this study, POP significantly inhibited the HFD-induced increase in Firmicutes abundance and reduced the *F*/*B* ratio, suggesting that POP’s regulation of gut microbiota helps alleviate HFD-induced obesity.

At the genus level, HFD altered the abundance of related bacteria. Our results show that HFD increased the abundances of *Helicobacter* and *Mucispirillum*, while decreasing those of *Parabacteroides* and *Muribaculaceae*. Growing evidence links *Helicobacter* and *Mucispirillum* to increased risk of NAFLD (27, 28). *Muribaculaceae* may be associated with resistance to HFD in lean mice and enhance the barrier function of the intestinal mucus layer (29). Additionally, the relative abundance of *Muribaculaceae* was significantly reduced in ApoE*^−/−^* mice fed HFD for 12 weeks, consistent with our current findings (30). *Parabacteroides* is reported to be less abundant in obese individuals and has shown anti-obesity effects in animal studies (31). Cuffaro et al. (32) found that two strains of *Parabacteroides distasonis* promoted the secretion of the incretin glucagon-like peptide-1 (GLP-1) *in vitro* and effectively inhibited weight gain and fat accumulation in an obese mouse model. LEfSe analysis showed that POP intervention significantly increased the abundances of beneficial bacteria such as *Parabacteroides*, *Acetatifactor*, and *Incertae_Sedis*. Time-restricted feeding (TRF), an effective dietary strategy for metabolic regulation by optimizing energy utilization, improving metabolic syndrome, and enhancing microbial circadian fluctuations (33), significantly improves obesity and NASH and restores the rhythmicity of *Acetatifactor* and other genera (33). Additionally, *Acetatifactor* abundance was specifically reduced in rodent models of depression (34). Studies have shown that apigenin intervention reduced body weight and lipid levels in obese mice while upregulating *Incertae_Sedis* abundance, suggesting that changes in *Incertae_Sedis* abundance are associated with obesity (35). *Anaerotruncus*, a butyrate producer, is increased in healthy individuals on a high saturated fat/low fiber diet. Its abundance progressively increases with the progression of fatty degeneration, steatohepatitis, and hepatocellular carcinoma (HCC) (28). Current research on the role of *Enterorhabdus* in specific diseases is limited. In a study on the anti-obesity effect of piperine, *Enterorhabdus* abundance was inhibited in obese mice with reduced body weight, serum TG, TC, LDL-C, and blood glucose levels (36). In our study, POP intervention reduced the abundances of *Enterorhabdus* and *Anaerotruncus*, indicating POP’s positive effects on reducing body weight, blood lipids, and glucose. Previous studies found reduced relative abundance of *Defluviitaleaceae UCG011* in HFD-induced hyperlipidemic rats (37). Similar to our results, *Defluviitaleaceae UCG011* and *Enterorhabdus* were enriched in 10 month-old female C57BL/6 mice fed HFD (45% kcal from fat) (38). These studies suggest that *Defluviitaleaceae UCG011* exhibits different characteristics in different rodent species on HFD. PICRUSt2 analysis showed significant differences in genes related to sulfur relay system, glycerolipid metabolism, porphyrin and chlorophyll metabolism, QS, ABC transporters, and other glycan degradation among the three groups (Figure 6H). A study exploring the association between healthy diets and microbiota found that Healthy Food Choices (HFC) scores were positively correlated with functions such as short-chain fatty acid metabolism and synthesis, and negatively correlated with functions like fatty acid biosynthesis and sulfur relay system (39). Dysregulation of glycerolipid metabolism is increasingly linked to metabolic disorders such as obesity, insulin resistance, and NAFLD (40). Porphyrin and chlorophyll metabolism are important metabolic pathways in organisms, and their dysfunction may be associated with hemoglobin synthesis disorders (41). QS plays a key role in gut microbiota homeostasis (42), and disrupting QS can imbalance gut microbiota and lead to diseases. As the largest superfamily of transport proteins widely present in organisms, ATP-binding cassette (ABC) transporters are mainly localized in lipid metabolism-related cells. Notably, their lipid transport properties make them critical in metabolic diseases (43). Interestingly, these metabolic pathway functions were significantly enhanced in long-term HFD-fed mice, while other glycan degradation functions were weakened. Therefore, POP may exert anti-obesity effects by reshaping gut microbiota homeostasis and related metabolic functions.

In this study, analysis of colonic content metabolite profiles showed that dietary changes induced metabolic shifts. Among differential metabolites, succinyladenosine, 9r,10s-Epome, LacCer (d18:1/12:0), and NDC were common differential metabolites among the three groups. 9r,10s-Epome and LacCer (d18:1/12:0) belong to Lipids and lipid-like molecules and are involved in lipid metabolism. Succinyladenosine is a purine nucleoside, and recent clinical trials have demonstrated significant therapeutic potential of purine nucleosides in cardiovascular and neurological diseases (44). NDC, with structural features of coumarin derivatives and benzodihydropyran derivatives, remains unclassified, and its role in POP’s anti-obesity effect requires further study. KEGG analysis showed that differential metabolites were involved in metabolic pathways among groups. Pathways such as linoleic acid metabolism, glycerophospholipid metabolism, biosynthesis of unsaturated fatty acids, alpha-linolenic acid metabolism, and arachidonic acid metabolism are closely related to lipid metabolism. Metabolites involved in these pathways, such as rumenic acid (RA), 9r,10s-Epome, alpha-linolenic acid, PC (P-18:1 (9Z)/0:0), PC (20:4 (5Z,8Z,11Z,14Z)/0:0), and 12s-Hpete, were significantly increased in the HFD group compared to the Con group (3.51, 1.18, 3.33, 3.87, 3.34, and 1.14 times higher, respectively; data not shown). Studies have found significantly increased 9r,10s-Epome in follicular fluid of polycystic ovary syndrome (PCOS) patients with phlegm-dampness type (45). Notably, PCOS, the most common endocrine disease in reproductive-aged women, is often associated with obesity and impairs reproductive health (46). RA has gained attention for its potential health benefits like anti-diabetes, but a HFD rich in RA not only failed to improve glucose tolerance in obese mice but also exacerbated hepatic steatosis (47). Polyunsaturated fatty acids (PUFAs) such as linolenic acid and arachidonic acid (AA) are converted into oxylipins by cyclooxygenases (COX-1, COX-2) and lipoxygenases (LOXs), significantly influencing the progression of neurodegenerative diseases (48). Thus, we speculate that HFD leads to excessive unsaturated fatty acids in obese individuals, which is detrimental to health. Compared to the HFD group, 9r,10s-Epome involved in linoleic acid metabolism was significantly reduced in the POP group (Figure 8B). The correlation analysis showed that 9r,10s-Epome was positively correlated with inguinal fat mass (Figure 9B). Additionally, metabolites involved in glycerolipid metabolism (dihydroxyacetone, DHA) were significantly increased in the POP group (1.36 times higher than HFD group; data not shown). DHA is an ATP energy source that significantly improves mitochondrial function (49). Based on these findings, our results suggest that POP may improve lipid metabolism and reduce fat accumulation by decreasing 9r,10s-Epome, LacCer (d18:1/12:0), and NDC, while increasing succinyladenosine and DHA biosynthesis.

Studies have identified *Anaerotruncus* and *Enterorhabdus* as major bacterial genera contributing to obesity (50, 51). Our study found that the harmful bacteria *Anaerotruncus* and *Enterorhabdus* were significantly positively correlated with obesity phenotypes and lipid level indicators (Figure 9A). *Parabacteroides* has physiological properties of carbohydrate metabolism and short-chain fatty acid production. Recent studies show this genus is closely associated with host health (e.g., metabolic syndrome, inflammatory bowel disease, and obesity) (52). In previous research, colonization of live *Parabacteroides merdae* in animals effectively reduced atherosclerotic plaques in HFD-fed ApoE-deficient male mice (53). Correlation analysis between obesity phenotype indicators and gut microbiota in this study showed that *Parabacteroides* had a strong negative correlation with liver weight and TG (Figure 9A). Notably, the liver plays a key role in regulating whole-body homeostasis and is a critical site for lipid metabolism (54). Additionally, LacCer (d18:1/12:0) and 9r,10s-Epome were significantly positively correlated with related obesity phenotype indicators (Figure 9B). These results may further indicate that POP alleviates host weight gain and abnormal glucose tolerance by increasing the abundance of beneficial probiotics and modulating changes in intestinal metabolites such as Succinyladenosine, 9r,10s-Epome, LacCer (d18:1/12:0), and NDC.

## Conclusion

This study confirms that POP supplementation alleviates obesity and regulates gut microbiota and metabolic functions in HFD mouse models. Specifically, POP significantly reduces body weight, hepatic lipid accumulation, and adipocyte hypertrophy in HFD mice. This intervention also improves glucose homeostasis regulation and effectively modulates serum lipid levels. Through gut microbiota analysis and metabolomics, POP was found to not only alter gut microbial community structure but also significantly regulate metabolite composition. This study systematically elucidates the anti-obesity mechanism of POP, providing important theoretical basis for its *in vivo* molecular regulation pathway.

## Data Availability

The data presented in the study are deposited in the NCBI SRA repository, accession number PRJNA1422999 (https://www.ncbi.nlm.nih.gov/sra/PRJNA1422999).

## Footnotes

1
https://cloud.oebiotech.com

2
http://www.genome.jp/kegg/

## Supplementary material

SUPPLEMENTARY TABLE S1 The composition of MD12032, MD12032+3.2%POP and MD12031.

SUPPLEMENTARY TABLE S2 Information of internal standards, solvents, and reagents.

SUPPLEMENTARY TABLE S3 Table of differential metabolites between the HFD group and the Con group.

SUPPLEMENTARY TABLE S4 Table of differential metabolites between the POP group and the HFD group.

SUPPLEMENTARY TABLE S5 Table of main metabolic pathways between the HFD group and the Con group.

SUPPLEMENTARY TABLE S6 Table of main metabolic pathways between the POP group and the HFD group.
