# Supplementary material for: Portulaca oleracea polysaccharide alleviates obesity in mice with long-term high-fat diet by regulating gut microbiota and metabolites
Source: Front Nutr. 2026 Feb 27;13:1759556. doi: 10.3389/fnut.2026.1759556 (PMC12982438; doi:10.3389/fnut.2026.1759556)
Supplement: SUPPLEMENTARY TABLE S1 — The composition of MD12032, MD12032+3.2%POP and MD12031. [file Table_1.doc]

Table S1. The composition of MD12032 , MD12032+3.2%POP and MD12031.

| Product# | MD12032 | MD12032+3.20%POP | MD12031 |
| --- | --- | --- | --- |
| Casein | 23.31% | 23.31% | 18.96% |
| Corn starch | 8.51% | 5.31% | 42.86% |
| Maltodextrin | 11.65% | 11.65% | 7.11% |
| Sucrose | 20.14% | 20.14% | 16.38% |
| Fibre | 5.83% | 5.83% | 4.74% |
| Soybean oil | 2.91% | 2.91% | 2.37% |
| Pork fat | 20.68% | 20.68% | 1.90% |
| *Purslane polysaccharide* | __ | 3.20% | __ |
| Complex minerals, vitamins, etc | 6.97% | 6.97% | 5.68% |
| Total | 100% | 100% | 100% |
