# Supplementary material for: Portulaca oleracea polysaccharide alleviates obesity in mice with long-term high-fat diet by regulating gut microbiota and metabolites
Source: Front Nutr. 2026 Feb 27;13:1759556. doi: 10.3389/fnut.2026.1759556 (PMC12982438; doi:10.3389/fnut.2026.1759556)
Supplement: SUPPLEMENTARY TABLE S2 — Information of internal standards, solvents, and reagents. [file Table_2.docx]

Supplementary Table S2. Information of internal standards, solvents, and reagents

| **Reagent Name** | **Catalog Number** | **Supplier** | **Purity** |
| --- | --- | --- | --- |
| Methanol | A452-4 | Thermo Fisher | HPLC Grade, 99.9% |
| Acetonitrile | A998-4 | Thermo Fisher | HPLC Grade, 99.95% |
| Formic Acid | A117-50 | Thermo Fisher | HPLC Grade, 99.0% |
| Pure Water | — | Wahaha Group Corporation, Hangzhou | — |
| L-2-Chlorophenylalanine (mixed internal standard) | C2001 | Shanghai Hengchuang Biotechnology Co., Ltd. | HPLC Grade, 98.0% |
| Succinic Acid-d4 (mixed internal standard) | 293075-1G | Sigma-Aldrich | HPLC Grade, 98.0% |
| L-Valine-d8 (mixed internal standard) | HY-I1124 | Shanghai Haoyuan Biotechnology Co., Ltd. | HPLC Grade, 98.0% |
| Cholic Acid-d4 (mixed internal standard) | S22155-50mg | Shanghai Yuanye Biotechnology Co., Ltd. | HPLC Grade, 98.0% |
